# Multimodal Assessment of Hand Hygiene Quality Using ATP Bioluminescence, Microbiological Culture, and UV-Fluorescence Digital Imaging: A Prospective Before–After Study Across Intensive Care, Hematology, and Gynecology Departments

**DOI:** 10.3390/jcm15124756

**Published:** 2026-06-18

**Authors:** Lucrețiu Radu, Marius-Bogdan Novac, Ramona-Constantina Vasile, Alexandra-Daniela Rotaru-Zăvăleanu, Liviu Martin, George-Alin Stoica

**Affiliations:** 1 Department of Hygiene, University of Medicine and Pharmacy of Craiova, 200349 Craiova, Romania; lucretiu.radu@umfcv.ro; 2 Department of Anesthesiology and Intensive Care, University of Medicine and Pharmacy of Craiova, 200349 Craiova, Romania; 3 Department of Epidemiology, University of Medicine and Pharmacy of Craiova, 200349 Craiova, Romaniaalexandra.rotaru@umfcv.ro (A.-D.R.-Z.); 4 Faculty of Medical Care, Titu Maiorescu University, Văcărești Road, No. 187, 040051 Bucharest, Romania; 5 Department of Pediatric Surgery, University of Medicine and Pharmacy of Craiova, 200349 Craiova, Romania; alin.stoica@umfcv.ro

**Keywords:** hand hygiene, ATP bioluminescence, microbiological monitoring, UV-fluorescence, Semmelweis system, healthcare-associated infections, infection prevention, before–after study, multimodal assessment

## Abstract

**Background**: Healthcare-associated infections (HAIs) remain a critical patient safety challenge. Hand hygiene is considered the most effective preventive measure, yet traditional monitoring captures only compliance, not technique quality. This prospective before–after study evaluated whether real-time visual feedback via the Semmelweis UV-fluorescence system is associated with improved hand hygiene quality, measured by ATP bioluminescence and microbiological culture. **Methods**: Three clinical departments (the Intensive Care Unit, Hematology, and Gynecology) at a Romanian tertiary hospital were purposively selected. Seventy-one healthcare workers (HCWs) were enrolled. The 12-week study comprised Phase 1 (baseline, weeks 1–4), Phase 2 (active intervention with Semmelweis feedback, weeks 5–8), a one-week washout (week 9), and Phase 3 (sustainability assessment, weeks 10–12). Paired ATP-CFU samples were collected weekly. Within-group comparisons used Kruskal–Wallis H tests with post hoc Dunn’s tests and Bonferroni correction. Secondary outcomes included Semmelweis global and zone-specific coverage and the correlation between subject-level Semmelweis coverage and ATP bioluminescence (Spearman’s rho). **Results**: A total of 781 paired ATP-CFU samples and 497 Semmelweis evaluations were analyzed. Mean ATP declined from 195.9 RLU at baseline to 148.2 RLU in Phase 2 (−24.4%) and 154.8 RLU in Phase 3 (−21.0%; Kruskal–Wallis H = 102.73, *p* < 0.001). CFU/mL declined from 84.8 to 66.2 (−21.9%) and 70.7 (−16.6%; H = 22.48, *p* < 0.001). Post hoc comparisons confirmed significant Phase 1 versus Phase 2 and Phase 1 versus Phase 3 differences for both markers (all *p* < 0.01), while Phase 2 versus Phase 3 was non-significant, indicating stabilization at an improved level. Subject-level Semmelweis coverage correlated negatively with ATP (rho = −0.665, 95% CI −0.778 to −0.510, *p* < 0.001), supporting construct validity at the operator level. Semmelweis global coverage was 93.1% (Phase 2) and 90.6% (Phase 3); interdigital spaces showed the highest inadequacy rate (73.9% protocol-based, 92.5% targeted). **Conclusions**: Real-time visual feedback via UV-fluorescence imaging was associated with significant and sustained improvements in hand hygiene quality beyond baseline. ATP, CFU, and Semmelweis assessments captured complementary, non-redundant dimensions, supporting multimodal evaluation. Interdigital spaces and fingertips remained persistent failure points requiring targeted educational reinforcement.

## 1. Introduction

Healthcare-associated infections (HAIs) remain a major challenge in modern clinical medicine, affecting an estimated 7% of hospitalized patients in high-income countries and up to 15.5% in low- and middle-income settings, with substantial economic burden [1,2]. In the European Union, the European Centre for Disease Prevention and Control (ECDC) estimates approximately 3.8 million HAI cases annually, with 37,000 attributable deaths and direct costs exceeding EUR 7 billion [3]. Romania reports HAI prevalence rates above the European mean in certain high-acuity settings, reflecting historical underinvestment in infection prevention infrastructure [4].

Hand hygiene is considered the most impactful and cost-effective single intervention within comprehensive strategies for preventing HAIs and interrupting pathogen transmission in healthcare settings. The WHO ‘Clean Care is Safer Care’ initiative designates hand hygiene as the cornerstone of infection control, supported by decades of evidence including the landmark Geneva program demonstrating 30–50% HAI reduction through multimodal promotion [5,6]. Despite this, observed compliance rates among healthcare workers (HCWs) typically range between 40% and 60%, with technique quality receiving far less attention [7,8].

A critical insight frequently overlooked is that performing hand hygiene does not guarantee adequate decontamination. A suboptimal technique leaves substantial regions inadequately covered, particularly interdigital spaces, fingertips, and subungual areas [9,10]. Direct observation, the compliance reference standard, cannot assess spatial coverage quality [11].

ATP bioluminescence provides rapid quantification of organic contamination (RLU within seconds), adapted from environmental surface monitoring to hand assessment, though optimal hand-surface thresholds remain debated [12,13]. Microbiological culture quantifies viable microbial burden but requires 24–48 h [14]. The Semmelweis Scanner uses UV-A excited fluorescence digital imaging to compute global and zone-specific antiseptic coverage, providing immediate individualized visual feedback [15,16]. These modalities probe non-overlapping dimensions: ATP measures total organic contamination, culture measures viable organisms, and Semmelweis maps spatial application technique. Throughout this work, we use the term “hand hygiene quality” to denote the multidimensional adequacy of hand decontamination, encompassing residual organic contamination (ATP bioluminescence), viable microbial burden (microbiological culture), and the spatial completeness of antiseptic application (UV-fluorescence coverage), as distinct from hand hygiene compliance, which captures only whether an event occurred.

Although the Semmelweis Scanner has been evaluated in pre–post designs [17], to our knowledge no prior study has deployed all three modalities within a prospective before–after design to assess the effect of visual feedback while characterizing inter-modality concordance. We report a three-phase prospective study across three clinical departments evaluating the association between the Semmelweis system and hand hygiene quality. The study focuses on proximal quality outcomes (organic contamination, microbial burden, and spatial coverage), rather than HAI incidence; the latter requires substantially larger samples and longer follow-up, and is addressed in a planned follow-up study.

We hypothesized that real-time individualized UV-fluorescence feedback would be associated with measurable improvements in hand hygiene quality—specifically, reductions in ATP bioluminescence and microbiological CFU and increases in antiseptic coverage, relative to baseline—and that these improvements would persist beyond the active feedback period. We further hypothesized that the three modalities would capture complementary, non-redundant dimensions of hand hygiene quality, rather than redundant signals of a single construct. Accordingly, our objectives were to (1) quantify phase-level change in ATP and CFU across baseline, active-intervention, and sustainability phases; (2) characterize global and zone-specific antiseptic coverage; and (3) assess the concordance among the three modalities.

## 2. Materials and Methods

### 2.1. Study Design

This was a prospective, three-phase before–after study conducted at the Clinical County Hospital of Craiova, Romania, affiliated with the University of Medicine and Pharmacy of Craiova, between 6 January 2026 and 30 March 2026. Three clinical departments (the Intensive Care Unit, Hematology, and Gynecology) were purposively selected to represent diverse clinical acuities and staffing intensities. Baseline department-level characteristics (mean ATP, mean CFU, HCW count, and observed compliance) were assessed and are reported in Table 1 and Table S1. This study is reported following the SQUIRE 2.0 (Standards for Quality Improvement Reporting Excellence) guidelines [18] (Supplementary Material S1). In line with SQUIRE 2.0 reporting expectations, potential sources of bias, the treatment of repeated measures, the rationale for outcome thresholds, and the generalizability of findings are addressed explicitly in the Methods (Section 2.4 and Section 2.6) and Discussion (Section 4.3 and Section 4.6).

**Figure 1:**
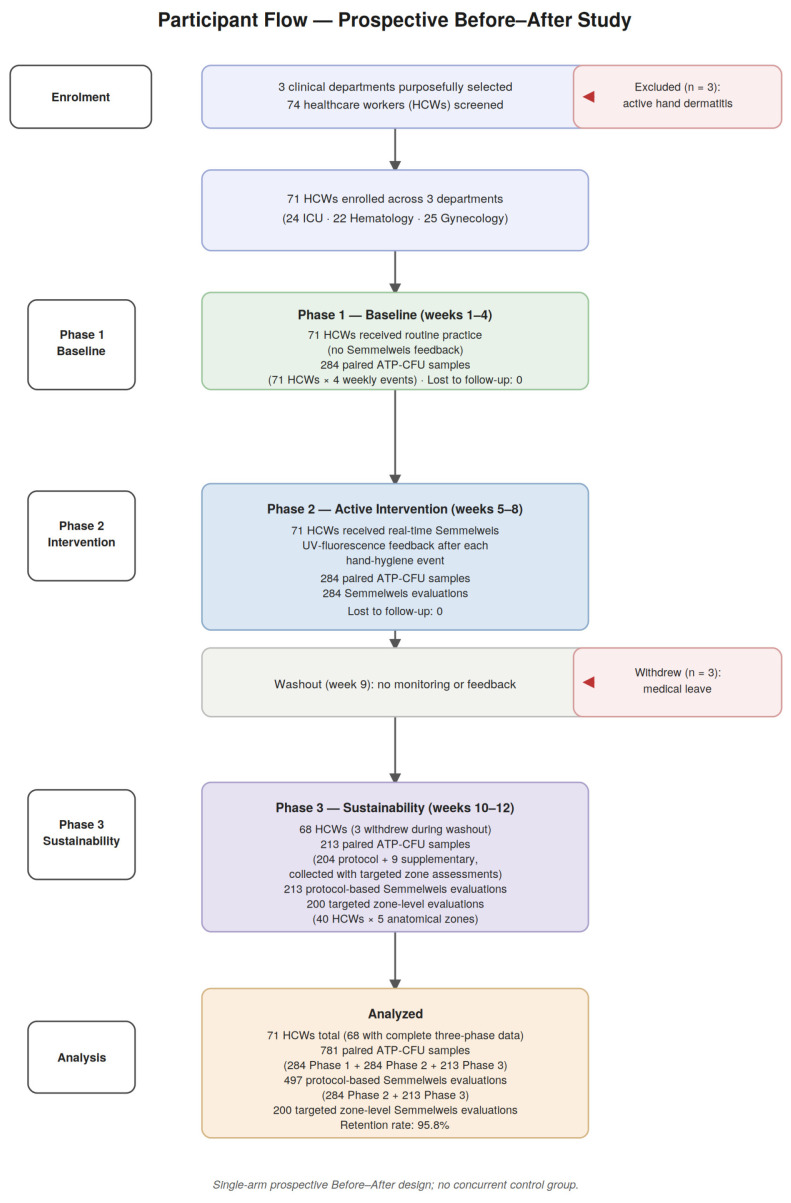
Participant flow diagram for the prospective Before–After study.

The study protocol was approved by the Ethics Committee of the University of Medicine and Pharmacy of Craiova (approval no. 29292/26 November 2025). All participants provided written informed consent. The participant flow is shown in Figure 1.

### 2.2. Participants

A total of 74 HCWs were screened for eligibility across the three departments; 3 were excluded due to active hand dermatitis. The remaining 71 HCWs were enrolled (24 ICU, 22 Hematology, and 25 Gynecology). The professional role distribution was the following: 12 attending physicians (16.9%), 3 medical residents (4.2%), 42 nurses (59.2%), and 14 nursing assistants (19.7%). Inclusion criteria: active clinical employment with regular direct patient contact; ability to participate across all study phases (Supplementary Table S1). Exclusion criteria: active hand dermatitis precluding alcohol-based rub use; planned absence exceeding two consecutive weeks. Three participants withdrew during the study due to medical leave, yielding a 95.8% retention rate. The full participant flow is shown in Figure 1, and baseline characteristics by department and role are presented in Table 1.

### 2.3. Study Phases and Sampling Schedule

Phase 1 (Baseline Assessment, Weeks 1–4): all participants performed routine hand hygiene, and sampling was standardized to a single WHO hand hygiene moment (Moment 1—before patient contact) across all three departments. This standardization of the sampling moment, rather than of the total number of hygiene opportunities, ensured that ATP and CFU samples were drawn from an equivalent hand hygiene context in every department, despite the inherently higher frequency of hygiene opportunities in higher-acuity settings such as the ICU. Samples were collected without disclosure of individual results. The Semmelweis Scanner was not used.

Phase 2 (Active Intervention, Weeks 5–8): participants received real-time individualized visual feedback via the Semmelweis Scanner after each hand hygiene event. Feedback consisted of a ~90-s review of color-coded hand coverage maps with corrective guidance. Participants were not informed of aggregate results during the active phase.

Washout (Week 9): No formal monitoring or feedback.

Phase 3 (Sustainability Assessment, Weeks 10–12): participants resumed ATP + CFU sampling. Additionally, targeted zone-specific Semmelweis assessments were performed. Forty HCWs were sampled, each evaluated separately at the five anatomical zones (palm/metacarpal, interphalangeal joints, fingertips, subungual area, and interdigital spaces), yielding 200 zone-level evaluations focused on regions of greatest concern identified in Phase 2.

Sampling schedule. Each enrolled HCW was scheduled for one paired ATP-CFU sampling event per week as the protocol baseline. Phases 1 and 2 (4 weeks each) yielded a maximum of 4 sampling events per HCW per phase, providing 284 paired samples per phase (71 × 4). In Phase 3 (3 weeks), three participants had withdrawn, leaving 68 HCWs. The base protocol prescribed three weekly sampling events per HCW, yielding 204 protocol-based samples. An additional 9 paired samples were collected from the same participants during their scheduled targeted zone-specific Semmelweis sessions; these were obtained for logistical convenience while participants were already present for assessment, and were not selected on the basis of any participant’s prior performance, contamination level, or coverage result. The final Phase 3 sample count was therefore 213 (204 protocol-based + 9 supplementary), yielding a total of 781 paired ATP-CFU samples: 284 (Phase 1) + 284 (Phase 2) + 213 (Phase 3). Because the supplementary samples constituted only 4.2% of Phase 3 observations (1.2% of the total dataset) and were not performance-selected, they are not expected to introduce directional bias or materially affect phase-level comparisons.

### 2.4. Assessment Methods

#### 2.4.1. ATP Bioluminescence

ATP was measured using a 3M Clean-Trace NG3 luminometer (3M Company, St. Paul, MN, USA) immediately following hand hygiene events, using a standardized swabbing technique applied to the entire palmar surface of the dominant hand, including the fingers and interdigital spaces, swabbed in a systematic serpentine pattern under uniform pressure. The luciferase-catalyzed reaction detects ATP from all biological material, expressed as Relative Light Units (RLUs). Because validated thresholds for ATP on hand surfaces are not formally standardized, we adopted operational categories adapted from environmental-surface literature [12] where ATP bioluminescence is well characterized as a relative index of total organic residue. These cut-points were used as internally consistent operational strata for comparative purposes across study phases, rather than as diagnostic standards of hand cleanliness; because all phases were assessed with identical thresholds and instrumentation, between-phase comparisons remain valid, irrespective of the absolute calibration of the cut-points.

Because no internationally validated RLU thresholds specific to hand surfaces have been established, the operational categories applied here (Optimal < 150 RLU, Marginal 150–300 RLU, Inadequate > 300 RLU) were adapted from the environmental-surface ATP monitoring literature [12], in which RLU benchmarks of this order have been used to stratify organic residue and define pass/caution/fail levels on the same 3M Clean-Trace platform employed in this study. We emphasize that these cut-points were not derived from validated hand-cleanliness standards, as none currently exist, but served as internally consistent operational strata for comparing organic contamination across study phases. Because all phases were assessed with identical thresholds, instrumentation, and calibration, between-phase comparisons remain valid, irrespective of the absolute calibration of the cut-points.

#### 2.4.2. Microbiological Culture

Hand surface samples were collected using pre-moistened sterile swabs immediately after ATP measurement from the same palmar surface immediately after ATP measurement. Samples were transferred within one hour, cultured on blood agar and MacConkey agar, incubated at 37 °C for 24–48 h, and expressed as CFU/mL. Classification thresholds were operationally defined consistent with prior healthcare-worker hand surveillance studies: Satisfactory (<50 CFU/mL), Marginal (50–200 CFU/mL), or Unsatisfactory (>200 CFU/mL); EN 1500:2013 [14] was used as the in vitro reference for handrub efficacy testing, not as a direct source of clinical CFU thresholds.

#### 2.4.3. Semmelweis UV-Fluorescence Digital Imaging

The HandInScan™ Semmelweis Scanner (HandInScan Zrt., Budapest, Hungary) captures UV-A (365 nm)–excited fluorescence from a proprietary fluorescent hand rub formulation [17]. Following 30 s standardized application per WHO guidelines, both hands were imaged. The system computes the global percentage of hand surface area covered by fluorescent hand antiseptic and zone-specific anatomical surface coverage across five predefined regions: palm/metacarpal, interphalangeal joints, fingertips, subungual area, and interdigital spaces. Coverage thresholds: Excellent (>95%), Acceptable (90–95%), Inadequate (<90%) [15,16].

#### 2.4.4. Outcome Assessment and Blinding

Outcome measurements were collected by two trained study personnel rotating across all three departments according to a pre-planned schedule. ATP luminometer readings were generated automatically by the 3M Clean-Trace device and recorded directly; the recorded RLU value was not subject to interpretive judgment. Microbiological cultures were processed and enumerated by two or more trained microbiologists at the Microbiology Department who were blinded to the sampling phase: samples were labelled with sequential study codes and the master coding key was held by the trial statistician and released only after database lock. To ensure counting reliability, inter-observer consistency was verified on a randomly selected subset of plates independently counted by a second microbiologist; agreement was high, and discrepancies were resolved by consensus re-count before values were entered into the database. Semmelweis evaluations were generated automatically by the device’s image-analysis algorithm.

### 2.5. Outcomes

Primary outcomes: (1) change in ATP bioluminescence (mean RLU) from baseline (Phase 1) to Phase 2 and Phase 3; and (2) change in CFU/mL along the same trajectory. Secondary outcomes: (3) Semmelweis global and zone-specific coverage; (4) correlation between subject-level Semmelweis coverage and ATP bioluminescence (Spearman’s rho); and (5) sustainability of improvements through the washout period.

### 2.6. Statistical Analysis

Analyses were conducted using MedCalc v22.015 (Ostend, Belgium). Normality was assessed via the Shapiro–Wilk test. Continuous variables are reported as mean +/− SD and median (interquartile range, IQR).

Primary inferential analysis. Within-group changes in ATP across the three study phases were assessed using the Kruskal–Wallis H test, with a post hoc Dunn test and Bonferroni correction for three pairwise comparisons (alpha = 0.017). For transparency, uncorrected Mann–Whitney U values are also reported. Within-group changes in CFU counts were assessed using the same non-parametric approach. These phase comparisons characterize group-level (phase-level) distributional differences across study phases; the analysis was not structured as a within-person paired design, and the findings should accordingly be interpreted as phase-level rather than individual-level change. We selected non-parametric phase-level tests (Kruskal–Wallis with post-hoc Dunn) because the primary inferential question concerned shifts in the population distribution of contamination markers between study phases, rather than within-individual change. We acknowledge that repeated sampling of the same HCWs introduces within-subject clustering that these tests do not explicitly model; consequently, the reported *p*-values may not fully account for within-subject correlation, and inference should be understood at the phase (population) level, rather than the level of individual hygiene events. Because the weekly sampling schedule, three withdrawals, and supplementary Phase 3 opportunistic sampling produced an unbalanced repeated-measures structure, a fully specified mixed-effects model was not pursued in the present analysis; this is identified as a methodological priority for the prospectively balanced follow-up study.

Sensitivity analysis. Because ATP RLU values exhibited right-skew, a sensitivity analysis was conducted on log-transformed ATP values using the same Kruskal–Wallis framework. Effect sizes for the overall phase effect were quantified using epsilon-squared (ε^2^), interpreted as small (0.01), medium (0.06), or large (0.14). For post hoc pairwise comparisons, effect sizes were expressed as r = Z/√N, with 95% confidence intervals derived via the Fisher z-transformation. The robustness of conclusions to distributional assumptions was confirmed by the concordance between original-scale and log-transformed analyses.

Secondary and descriptive analyses. Hand laterality was assessed via the Wilcoxon signed-rank test. Zone-specific Semmelweis coverage was compared via the Friedman test. Associations between Semmelweis coverage and ATP bioluminescence used Spearman’s rank correlation, with subject-level aggregation (one mean value per HCW) clearly distinguished from sample-level analyses in the text. Statistical significance was set at two-tailed *p* < 0.05.

Sample size. The study was designed to detect a 20% reduction in mean ATP RLU between baseline (Phase 1) and the active intervention phase (Phase 2). Assuming a within-subject standard deviation of approximately 58 RLU and a correlation of 0.5 between repeated measures on the same HCW, a paired-sample *t*-test framework yielded a required sample size of approximately 36 participants for 80% power at α = 0.05. Accounting for 15% anticipated attrition, the target enrolment was 42 participants; the final enrolment of 71 HCWs across three departments substantially exceeded this target.

Although the a priori sample-size calculation was framed within a paired-sample t-test paradigm, the conventional parametric reference for power estimation, the final analyses employed non-parametric tests (Kruskal–Wallis and Mann–Whitney) after the Shapiro–Wilk test indicated departures from normality. This does not compromise statistical power: non-parametric rank-based tests retain approximately 95% asymptotic relative efficiency relative to their parametric counterparts under normality, and their efficiency can equal or exceed that of the t-test for the right-skewed distributions observed here. Moreover, the final enrolment (71 HCWs; ~284 samples per phase) substantially exceeded the 36 participants required by the parametric calculation, providing a wide power margin, even after accounting for any efficiency adjustment. The parametric framework was therefore retained for transparency of the original design, while the non-parametric tests were selected post hoc to match the observed data distribution.

## 3. Results

### 3.1. Participant Characteristics and Sample Distribution

A total of 71 HCWs contributed 781 paired ATP–CFU samples across three phases (Phase 1: 284; Phase 2: 284; Phase 3: 213). Additionally, 497 protocol-based Semmelweis evaluations were performed (284 in Phase 2, 213 in Phase 3), plus 200 targeted zone-level Semmelweis evaluations in Phase 3 (40 HCWs × 5 anatomical zones). Three participants withdrew (4.2%), yielding 95.8% retention. Baseline characteristics by department and role are presented in Table 1 and Figure 1.

### 3.2. ATP Bioluminescence

**Figure 2:**
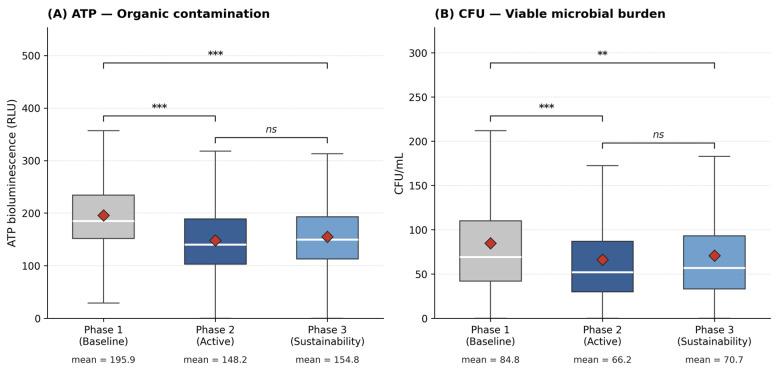
Distribution of ATP bioluminescence (**A**) and CFU/mL (**B**) across the three study phases. Boxplots show median (white line), IQR (box), and Tukey 1.5 × IQR whiskers. Mean values are indicated by red diamonds. Significance markers from post hoc Mann–Whitney U comparisons with Bonferroni correction: *** *p* < 0.001, ** *p* < 0.01, ns = not significant. Sample sizes: 284 (Phase 1), 284 (Phase 2), 213 (Phase 3).

ATP bioluminescence declined significantly across the three phases (Kruskal–Wallis H = 102.73, *p* < 0.001). Mean RLU decreased from 195.9 +/− 58.3 at baseline to 148.2 +/− 58.3 during the active intervention phase (−24.4%), and to 154.8 +/− 56.5 during the sustainability phase (−21.0% from baseline), consistent with durable improvement. ATP and CFU results by study phase are summarized in Table 2. Post hoc pairwise comparisons demonstrated significant differences between Phase 1 and Phase 2 (Mann–Whitney U = 58,740, *p* < 0.001) and between Phase 1 and Phase 3 (U = 42,234, *p* < 0.001), while Phase 2 versus Phase 3 was not significant (U = 27,556, *p* = 0.090), indicating stabilization at an improved level (Figure 2A). Effect sizes for all pairwise comparisons are summarized in Table 3.

The overall phase effect was large (ε^2^ = 0.132). Post hoc effect sizes confirmed substantial Phase 1 versus Phase 2 (r = 0.40, 95% CI 0.32–0.46) and Phase 1 versus Phase 3 (r = 0.34, 95% CI 0.26–0.42) differences, whereas the Phase 2 versus Phase 3 effect was negligible (r = 0.08, 95% CI −0.01 to 0.16), quantitatively corroborating stabilization at an improved level.

### 3.3. Microbiological Culture Results

Microbiological results paralleled the ATP trajectory. Mean CFU/mL declined significantly across phases (H = 22.48, *p* < 0.001): from 84.8 +/− 60.5 at baseline to 66.2 +/− 53.1 in Phase 2 (−21.9%), and to 70.7 +/− 53.3 in Phase 3 (−16.6%). Post hoc comparisons confirmed significant Phase 1 versus Phase 2 (U = 49,323, *p* < 0.001) and Phase 1 versus Phase 3 (U = 35,153, *p* = 0.002) differences, while Phase 2 versus Phase 3 did not reach significance, indicating sustained improvement (Figure 2B).

The rate of Unsatisfactory CFU results (>200 CFU/mL) decreased from 5.3% at baseline to 3.5% in Phase 2 and 2.8% in Phase 3. Satisfactory results (<50 CFU/mL) increased from 35.6% to 47.9% and 44.6%, respectively.

The overall phase effect for CFU was smaller (ε^2^ = 0.029). Post hoc effect sizes were r = 0.19 (95% CI 0.11–0.27) for Phase 1 versus Phase 2 and r = 0.14 (95% CI 0.05–0.22) for Phase 1 versus Phase 3, consistent with the more attenuated microbiological response discussed in Section 4.2.

### 3.4. Categorical ATP Distribution

The proportion of Optimal ATP readings (<150 RLU) increased substantially from 25.0% at baseline to 57.0% in Phase 2 and 51.2% in Phase 3. Inadequate readings (>300 RLU) decreased from 4.2% to 1.1% and 1.9%. The overall Phase 1 to Phase 3 shift was statistically significant (chi2 = 36.52, *p* < 0.001).

### 3.5. Semmelweis Coverage Analysis

Global hand coverage was 93.1 ± 4.3% during Phase 2 and 90.6 ± 4.3% during Phase 3, a modest decline of 2.5 percentage points (Mann–Whitney U = 31,876, *p* = 0.008). Notably, this small reduction in coverage occurred against stable Phase 3 ATP and CFU values. Several non-mutually-exclusive factors plausibly account for this pattern, including the non-linear relationship between coverage and contamination near the upper end of the coverage scale, the partial reintroduction of more challenging anatomical zones into Phase 3 assessment, and the absence of ongoing real-time feedback during the sustainability phase, each of which is examined in detail in the Discussion (Section 4.3). Coverage was classified as Excellent (>95%) in 27.4% of assessments, Acceptable (90–95%) in 39.6%, and Inadequate (<90%) in 33.0%.

A significant laterality effect was observed: the non-dominant (left) hand demonstrated higher mean coverage (93.2%) compared with the dominant (right) hand (90.7%), with a mean difference of 2.5 percentage points (Wilcoxon W = 97, *p* < 0.001).

**Figure 3:**
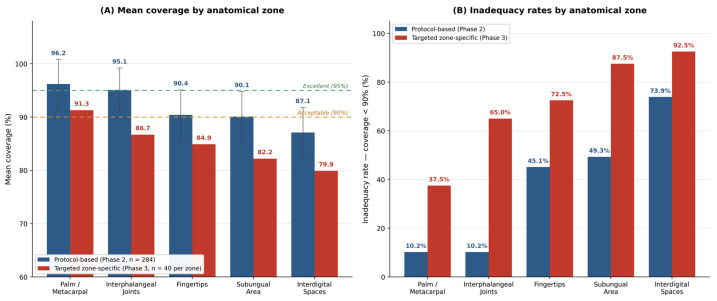
Semmelweis coverage by anatomical zone. (**A**) Mean coverage % comparing protocol-based assessments (Phase 2; n = 284 evaluations, ±1 SD error bars) and targeted zone-specific assessments (Phase 3; n = 40 HCWs per zone). Dashed lines indicate Excellent (≥95%) and Acceptable (≥90%) coverage thresholds. (**B**) Inadequacy rates (coverage < 90%) for both assessment modes. Targeted assessment consistently revealed substantially higher inadequacy than protocol-based aggregate evaluation, with interdigital spaces showing the highest failure rates.

Zone-specific analysis revealed highly significant differences in coverage across anatomical regions (Friedman chi2 = 450.12, *p* < 0.001). The palm/metacarpal region demonstrated the highest mean coverage (96.2%), followed by interphalangeal joints (95.1%), fingertips (90.4%), subungual area (90.1%), and interdigital spaces (87.1%), which had the lowest coverage. Inadequacy rates (<90%) ranged from 10.2% for the palm to 73.9% for interdigital spaces in protocol-based assessments. The differential between protocol-based and targeted zone-specific assessment is visualized in Figure 3, demonstrating that aggregate protocol evaluations substantially underestimate localized failure rates, particularly at distal anatomical zones.

Targeted zone-level assessments (Phase 3, n = 40 HCWs evaluated at each of the 5 zones) revealed substantially higher inadequacy rates than protocol-based estimates, with interdigital spaces reaching 92.5% and fingertips 72.5%, suggesting that focused evaluation captures technique failures obscured by aggregate assessments (Table 4).

### 3.6. Correlation Between Semmelweis Coverage and Contamination Markers

To reduce within-subject sampling noise, a subject-level analysis was performed in which each HCW’s measurements were aggregated to a single mean value of global Semmelweis coverage and of ATP RLU across Phases 2 and 3. Spearman analysis on the resulting 71 subject-level paired observations revealed a moderate negative correlation between global Semmelweis coverage and ATP bioluminescence (ρ = −0.665, 95% CI −0.778 to −0.510, *p* < 0.001), indicating that higher antiseptic coverage was associated with lower organic contamination. We emphasize that this is a subject-level (aggregated) correlation: each HCW contributed a single mean coverage value and a single mean ATP value, and the analysis therefore characterizes the between-operator association, rather than the within-event relationship. Because aggregation reduces measurement noise, subject-level correlations are systematically stronger than the corresponding sample-level associations; this is a recognized form of ecological inference, and the coefficient should not be extrapolated to the level of individual hygiene events. We accordingly interpret ρ = −0.665 as evidence of construct validity at the operator level only, and we explicitly caution against reading it as an event-level effect size. The direction of the association (higher coverage, lower contamination) is, however, biologically coherent and consistent across both the aggregated and the qualitative sample-level patterns.

### 3.7. Subgroup Analyses

Statistically significant differences in ATP bioluminescence were observed among clinical departments (Kruskal–Wallis H = 8.57, *p* = 0.014) but with small effect magnitude. Professional role showed a significant association with ATP values (H = 16.49, *p* < 0.001), with physicians demonstrating marginally lower mean RLU than nurses and nursing assistants. CFU values differed by department (H = 7.43, *p* = 0.024) but not by role (H = 5.96, *p* = 0.051). These differences were minor in absolute terms; sensitivity analyses stratified by department and role did not modify the magnitude or direction of the within-subject phase effects on either ATP or CFU.

## 4. Discussion

### 4.1. Principal Findings

This prospective before–after study provides multimodal evidence that real-time individualized visual feedback via UV-fluorescence imaging is associated with improvements in hand hygiene quality beyond standard monitoring alone. The 24.4% reduction in ATP bioluminescence and 21.9% reduction in CFU/mL during the active intervention represent substantial reductions in contamination markers. Because the study did not measure clinical infection endpoints, we refrain from characterizing these magnitudes as clinically meaningful in the sense of demonstrated patient-outcome benefit; rather, they indicate meaningful improvement in hand hygiene quality as captured by our proximal measures. These differences were supported by non-parametric testing (Kruskal–Wallis H = 102.73 for ATP, H = 22.48 for CFU; both *p* < 0.001), with post hoc comparisons demonstrating significant Phase 1 versus Phase 2 and Phase 1 versus Phase 3 differences (effect sizes reported in Table 3). The absence of significant Phase 2 versus Phase 3 differences indicates stabilization at an improved level rather than progressive decline, a pattern consistent with durable improvement.

The before–after design, while unable to fully exclude temporal confounding, incorporated several features that partially address alternative explanations: (a) the three-phase structure with an embedded washout week tests whether improvements persist in the absence of ongoing feedback; (b) the use of three independent assessment modalities (ATP, CFU, and Semmelweis) provides convergent measurement; and (c) the sustained concordance between ATP and CFU trajectories across all phases reduces the likelihood of measurement artifact at a single modality. The observation that improvements persisted through the washout period is consistent with technique internalization, rather than transient performance inflation, although the present design cannot directly confirm the underlying behavioral mechanism.

### 4.2. Interpretation of the Magnitude and Persistence of Improvement

The magnitude of improvement observed during the active feedback phase (mean within-subject reduction of 47.7 RLU for ATP, corresponding to a 24.4% decrease from baseline) is substantially larger than typical monitoring-alone effects reported in the hand hygiene literature, where passive observation typically yields 5–15% improvement. While suggestive, the absence of a concurrent control group within the present study precludes definitive attribution to the visual feedback per se, as opposed to other contributors (the Hawthorne effect, secular improvement, and study-period attention). The persistence of improvements through the washout phase (Section 4.1) provides indirect support for genuine behavioral change, rather than transient monitoring effects. Although the present study did not directly measure behavioral or cognitive processes, the observed improvements are plausibly mediated by several mechanisms proposed in the behavior-change literature: (1) translating abstract technique recommendations into personally salient, specific behavioral targets; (2) immediate temporal coupling of feedback to behavior (within seconds), consistent with reinforcement-learning principles; and (3) the visual impact of seeing objective coverage deficits, which may trigger emotional and motivational responses [19,20]. These mechanisms are offered as hypotheses consistent with our findings and the existing literature, rather than as pathways established by the present design; their direct assessment, for example, through validated behavioral or motivational measures, remains an objective for future work.

The sustainability of ATP improvements through the washout period (−21.0% maintained at three weeks post-intervention) suggests genuine internalization of technique refinements, rather than transient performance inflation. The more attenuated CFU sustainability (−16.6% versus −21.0%) likely reflects the greater ecological sensitivity of microbial burden to environmental recontamination and resident flora dynamics, which are less directly governed by technique alone.

We acknowledge that the absence of a concurrent control group limits our ability to exclude secular trends, seasonal effects, or temporal confounding, as alternative explanations. The study was conducted over a 12-week period (January–March), during which no hospital-wide hand hygiene campaigns or policy changes occurred; nevertheless, the Hawthorne effect of repeated sampling cannot be fully excluded. These limitations are addressed further in Section 4.6.

### 4.3. The Phase 3 Discordance Between Coverage and Contamination Markers

An apparent discordance was observed in Phase 3 between Semmelweis coverage and the contamination markers: global coverage declined modestly (93.1% to 90.6%, *p* = 0.008), while ATP and CFU remained essentially unchanged from Phase 2. Several non-mutually-exclusive explanations merit consideration. First, the relationship between coverage and contamination is likely non-linear: a 2.5 percentage-point change at the upper end of the coverage scale (close to the Excellent threshold) plausibly produces a smaller marginal change in contamination than would an equivalent decline at lower coverage levels. Second, HCWs may have differentially retained the technique elements most consequential for contamination reduction (e.g., palm and finger-pad contact during application) while regressing on technique elements that affect coverage scores but contribute less to organic burden reduction (e.g., the most distal fingertip and subungual coverage). Third, the Semmelweis system captures a single standardized application event under controlled imaging conditions, and may not fully reflect real-world technique during routine clinical work; the contamination markers, by contrast, integrate the actual recently-performed hygiene event, regardless of imaging conditions. Fourth, the targeted zone-specific assessments introduced in Phase 3 (Section 2.3) may have shifted the composition of measured events toward more challenging zones, contributing to lower aggregate coverage without a parallel contamination effect. The discordance cautions against treating Semmelweis coverage as a perfect surrogate for contamination outcomes.

The moderate Spearman correlation between subject-level Semmelweis coverage and ATP (ρ = −0.665, 95% CI −0.778 to −0.510, *p* < 0.001) is stronger than typical sample-level correlations reported in the literature, primarily because subject-level aggregation reduces measurement noise; this should not be misread as a stronger event-level association than has been previously demonstrated.

### 4.4. Anatomical Targeting: The Interdigital Space Problem

The interdigital spaces emerged as the most persistently undertreated region, with 73.9% inadequacy in protocol-based assessments and 92.5% in targeted evaluations. This magnitude—implying that more than nine in ten hand hygiene events inadequately cover this critical zone, even among HCWs receiving active feedback—warrants serious attention. The biomechanical challenge of the WHO Step 4 interlocking motion, easily abbreviated under time pressure, likely explains this persistence [21].

The fingertip coverage failure (72.5% inadequacy under targeted evaluation) similarly reflects the biomechanical difficulty of the WHO Step 5 interlocking-and-rotating motion. That these failures persist despite Semmelweis feedback suggests that single-session feedback may be insufficient to overcome deeply entrenched motor habits. Repeated feedback protocols or just-in-time reminders at dispensing points may be necessary [22].

### 4.5. Laterality Effects

The 2.5 percentage-point deficit on the dominant hand (*p* < 0.001) is consistent with prior UV-fluorescence assessments of hand antisepsis technique [17] and has an intuitive biomechanical explanation: the dominant hand serves as the active applicator and so receives less thorough opposing-hand contact during the application sequence. Given that the dominant hand typically performs procedural tasks, this systematic deficit carries direct patient safety implications, and should be explicitly addressed in training curricula.

### 4.6. Limitations

First, the single-arm before–after design without a concurrent control group is the most important methodological limitation. Although we incorporated a washout week and three-phase structure to partially address temporal confounding, we cannot exclude secular trends, seasonal variation, or the Hawthorne effect of repeated sampling as contributors to the observed improvements. The 12-week study duration (January–March) coincided with no hospital-wide hand hygiene campaigns or policy changes, which supports, but does not prove, attribution to the Semmelweis feedback itself. We also acknowledge that participation in the study, with weekly ATP-CFU sampling and immediate visual feedback during Phase 2, may itself have contributed to a Hawthorne-type performance effect that cannot be fully disentangled from the specific intervention.

Second, participant blinding to the intervention was not feasible because of the nature of the intervention: HCWs necessarily viewed their own UV-fluorescence images during Phase 2. Outcome assessor blinding was preserved for the CFU endpoint (laboratory personnel processed coded samples without phase knowledge; see Section 2.4.4) but not for ATP collection, where the visible presence of the Semmelweis Scanner during Phase 2 made operator blinding infeasible. ATP readings are nevertheless automated and not interpretively scored, limiting the scope for detection bias.

Third, the 12-week follow-up precludes conclusions about long-term durability beyond three months. Fourth, targeted zone assessments (n = 40 HCWs evaluated at each of the five zones) provide moderate precision; future studies should increase the targeted sample. Fifth, the single-center design and purposive selection of departments constrain external validity in several specific ways. The three departments (ICU, Hematology, and Gynecology) were chosen to represent contrasting clinical acuities and staffing intensities; while this strengthens internal heterogeneity, it does not constitute a representative sample of hospital units, and settings with markedly different baseline hygiene cultures, workloads, or staff-to-patient ratios may respond differently to visual feedback. Furthermore, the study was conducted at a single Romanian tertiary hospital with its own infection-prevention infrastructure, and the magnitude of improvement observed may not transfer directly to settings with higher baseline compliance, where the ceiling for improvement is lower, or to resource-limited settings lacking the Semmelweis equipment. Our cohort was also predominantly nursing staff (59%), leaving the response among other professional groups uncharacterized. We therefore consider these findings hypothesis-generating with respect to broad applicability: the consistent, convergent improvement across three heterogeneous departments supports the plausibility of transferable benefit, but multi-center studies across diverse institutional contexts and baseline hygiene cultures are required before extrapolation to other healthcare settings can be made with confidence. Sixth, the study assessed proximal quality outcomes (organic contamination, microbial burden, and spatial coverage), rather than HAI incidence; a clinically powered HAI-endpoint trial would require substantially larger samples and longer follow-up. Seventh, full pathogen-level characterization (species identification and antimicrobial susceptibility) of CFU isolates was performed but is reported in a separate companion manuscript currently under review. Eighth, our primary analyses compared phase-level distributions, rather than within-person trajectories. Because weekly sampling produced an unbalanced repeated-measures structure across participants, we did not perform a within-subject paired analysis or a mixed-effects model accounting for clustering by participant; the phase-level tests employed do not explicitly estimate within-subject correlation. Consequently, our findings reflect phase-level trends, rather than direct within-individual change. A paired design with complete repeated observations per participant, analyzed using Wilcoxon signed-rank or Friedman tests, would be required to formally confirm individual-level improvement, and this is planned for our follow-up study.

Several limitations of ATP bioluminescence for hand hygiene assessment warrant emphasis. ATP measures total organic residue, including non-microbial biological material such as skin cells, food debris, and cosmetic residues, and therefore does not directly quantify viable microbial burden; this is precisely why we paired ATP with microbiological culture as a complementary, non-redundant measure. RLU readings are further influenced by swab saturation, device-specific calibration, and surface moisture, limiting cross-study comparability of absolute values. Critically, no internationally validated RLU thresholds exist for hand surfaces, and our operational categories should not be interpreted as clinical decontamination standards. These considerations reinforce our multimodal approach and caution against reliance on ATP as a stand-alone surrogate for hand hygiene adequacy.

### 4.7. Surrogate Outcomes and Clinical Relevance

Our outcomes—ATP bioluminescence, microbiological CFU, and antiseptic coverage—are proximal surrogate markers of hand hygiene quality, rather than direct measures of healthcare-associated infection. The link between these markers and HAI reduction is biologically plausible and supported by the broader hand hygiene literature: viable microbial burden on hands is a recognized vector for cross-transmission, and multimodal hand hygiene promotion has been associated with 30–50% HAI reductions in landmark programs [5,6]. However, this chain of inference, from improved technique, to reduced hand contamination, to reduced transmission, to fewer HAIs, involves multiple steps that our design did not measure. CFU quantifies viable organisms most directly relevant to transmission risk, whereas ATP and coverage are one step further removed. We therefore interpret our findings as evidence of improved hand hygiene quality, not as direct evidence of reduced infection. Demonstrating an HAI-incidence effect would require a substantially larger, longer, and, ideally, controlled trial, which we have identified as a planned follow-up.

## 5. Conclusions

This prospective before–after study provides evidence that real-time individualized visual feedback via the Semmelweis UV-fluorescence system is associated with significant and sustained improvements in hand hygiene quality. The 24.4% reduction in ATP bioluminescence and 21.9% reduction in CFU/mL, maintained through a washout period, support the system’s potential utility as a training and quality improvement tool.

The moderate subject-level Semmelweis–ATP correlation (rho = −0.665), together with the Phase 3 discordance between coverage and contamination markers, indicates that these modalities capture complementary, non-redundant dimensions of hand hygiene quality. This reinforces the case for multimodal rather than single-modality assessment in infection prevention programs.

Interdigital spaces and fingertips remain critical anatomical failure points requiring targeted, repeated educational intervention. The absence of a concurrent control group limits causal inference, and controlled studies with larger samples and longer follow-up are warranted. Pending such evidence, the integration of visual feedback systems into routine infection prevention programs merits further evaluation in settings where technique quality, not merely compliance, may contribute to HAI risk.

## Figures and Tables

**Table 1:** Distribution of participants by department and professional role (N = 71).

Role	ICU	Hematology	Gynecology	Total
Attending physician	4	4	4	12
Medical resident	1	1	1	3
Nurse	14	13	15	42
Nursing assistant	5	4	5	14
Total	24	22	25	71

Column totals correspond to all enrolled HCWs; three participants subsequently withdrew due to medical leave.

**Table 2:** ATP bioluminescence and microbiological culture results by study phase.

Parameter	Phase	Mean ± SD	Median (IQR)	n
ATP (RLU)	1 (Baseline)	195.9 ± 58.3	184.8 (152–234)	284
	2 (Active)	148.2 ± 58.3	140.1 (103–189)	284
	3 (Sustain)	154.8 ± 56.5	149.3 (113–193)	213
CFU/mL	1 (Baseline)	84.8 ± 60.5	69.1 (42–110)	284
	2 (Active)	66.2 ± 53.1	52.0 (30–87)	284
	3 (Sustain)	70.7 ± 53.3	56.9 (33–93)	213

Effect sizes for post hoc pairwise comparisons (r = Z/√N; 95% CI via Fisher z-transformation): ATP—Phase 1 vs. 2, r = 0.40 (0.32–0.46), *p* < 0.001; Phase 1 vs. 3, r = 0.34 (0.26–0.42), *p* < 0.001; Phase 2 vs. 3, r = 0.08 (−0.01 to 0.16), *p* = 0.090 (ns). CFU—Phase 1 vs. 2, r = 0.19 (0.11–0.27), *p* < 0.001; Phase 1 vs. 3, r = 0.14 (0.05–0.22), *p* = 0.002. Overall phase effect (Kruskal–Wallis): ATP ε^2^ = 0.132 (large); CFU ε^2^ = 0.029 (small–medium).

**Table 3:** Effect sizes and confidence intervals for post hoc pairwise comparisons of contamination markers across study phases.

Comparison	Marker	r (95% CI)	*p*
Phase 1 vs. Phase 2	ATP	0.40 (0.32–0.46)	<0.001
Phase 1 vs. Phase 3	ATP	0.34 (0.26–0.42)	<0.001
Phase 2 vs. Phase 3	ATP	0.08 (−0.01 to 0.16)	0.090 (ns)
Phase 1 vs. Phase 2	CFU	0.19 (0.11–0.27)	<0.001
Phase 1 vs. Phase 3	CFU	0.14 (0.05–0.22)	0.002

Effect size r = Z/√N; 95% confidence intervals derived via Fisher z-transformation. Overall phase effect (Kruskal–Wallis): ATP ε^2^ = 0.132 (large); CFU ε^2^ = 0.029 (small–medium). ε^2^ interpreted as small (0.01), medium (0.06), large (0.14). ns = not significant.

**Table 4:** Zone-specific Semmelweis coverage from protocol-based (Phase 2) and targeted (Phase 3) assessments.

Anatomical Zone	Protocol Mean	Protocol SD	Protocol Inad. %	Targeted Mean	Targeted Inad. %
Palm/Metacarpal	96.2%	4.6%	10.2%	91.3%	37.5%
Interphalangeal Joints	95.1%	4.1%	10.2%	86.7%	65.0%
Fingertips	90.4%	4.7%	45.1%	84.9%	72.5%
Subungual Area	90.1%	4.7%	49.3%	82.2%	87.5%
Interdigital Spaces	87.1%	4.7%	73.9%	79.9%	92.5%

## Data Availability

The datasets analyzed during this study are available from the corresponding author upon reasonable request.

## Supplementary Materials

The following supporting information can be downloaded at: https://www.mdpi.com/article/10.3390/jcm15124756/s1, Supplementary Material S1: SQUIRE 2.0 Checklist [18]; Supplementary Table S1: Department-level distribution of healthcare workers and study samples.

## Abbreviations

The following abbreviations are used in this manuscript:

Abbreviation	Full term
ATP	Adenosine Triphosphate
CFU	Colony-Forming Units
CI	Confidence Interval
df	Degrees of Freedom
ECDC	European Centre for Disease Prevention and Control
EN	European Norm
HAI	Healthcare-Associated Infection
HCW	Healthcare Worker
ICU	Intensive Care Unit
IQR	Interquartile Range
RLU	Relative Light Units
SD	Standard Deviation
UV	Ultraviolet
WHO	World Health Organization
