# Supplementary material for: Multimodal Assessment of Hand Hygiene Quality Using ATP Bioluminescence, Microbiological Culture, and UV-Fluorescence Digital Imaging: A Prospective Before–After Study Across Intensive Care, Hematology, and Gynecology Departments
Source: J Clin Med. 2026 Jun 18;15(12):4756. doi: 10.3390/jcm15124756 (PMC13302134; doi:10.3390/jcm15124756)
Supplement: Supplementary file 1 [file jcm-15-04756-s001.zip › jcm-4352357-supplementary.pdf]

Supplementary Materials

Multimodal Assessment of Hand Hygiene Quality Using ATP Bioluminescence, Microbiological Culture, and UV-Fluorescence Digital Imaging: A Prospective Before-After Study Across Intensive Care, Hematology, and Gynecology Departments

Supplementary Material S1. SQUIRE 2.0 Checklist

This checklist follows the SQUIRE 2.0 (Standards for QUality Improvement Reporting Excellence) guidelines [18]

Page/section references correspond to the present manuscript. Each SQUIRE 2.0 item is mapped to the manuscript section(s) where the relevant content is reported.

Item	Section / Topic	SQUIRE 2.0 Item Description	Reported on Page / Section
**1**	Title and Abstract — Title	Indicate that the manuscript concerns an initiative to improve healthcare (broadly defined to include the quality, safety, effectiveness, patient-centeredness, timeliness, cost, efficiency, and equity of healthcare).	Title ("Multimodal Assessment of Hand Hygiene Quality... A Prospective Before-After Study")
**2**	Title and Abstract — Abstract	Provide adequate information to aid in searching and indexing. Summarize all key information from various sections of the text using the abstract format of the intended publication or a structured summary such as: background, local problem, methods, interventions, results, conclusions.	Abstract
**3**	Introduction — Problem Description	Nature and significance of the local problem.	Section 1 (Introduction), paragraphs 1–3
**4**	Introduction — Available knowledge	Summary of what is currently known about the problem, including relevant previous studies.	Section 1 (Introduction), paragraphs 4–5
**5**	Introduction — Rationale	Informal or formal frameworks, models, concepts, and/or theories used to explain the problem, any reasons or assumptions that were used to develop the intervention(s), and reasons why the intervention(s) was expected to work.	Section 1 (Introduction), final paragraph; Section 4.2 (Discussion)
**6**	Introduction — Specific aims	Purpose of the project and of this report.	Section 1 (final paragraph); Section 2.5 (Outcomes)
**7**	Methods — Context	Contextual elements considered important at the outset of introducing the intervention(s).	Section 2.1 (Study Design); Section 2.2 (Participants)
**8**	Methods — Intervention(s)	Description of the intervention(s) in sufficient detail that others could reproduce it. Specifics of the team involved in the work.	Section 2.3 (Study Phases); Section 2.4 (Assessment Methods, especially 2.4.3 Semmelweis system)

Item	Section / Topic	SQUIRE 2.0 Item Description	Reported on Page / Section
**9**	Methods — Study of the intervention(s)	Approach chosen for assessing the impact of the intervention(s). Approach used to establish whether the observed outcomes were due to the intervention(s).	Section 2.5 (Outcomes); Section 2.6 (Statistical Analysis); Section 4.1 (Discussion)
**10**	Methods — Measures	Measures chosen for studying processes and outcomes of the intervention(s), including rationale for choosing them, their operational definitions, and their validity and reliability. Description of the approach to the ongoing assessment of contextual elements that contributed to the success, failure, efficiency, and cost. Methods employed for assessing completeness and accuracy of data.	Section 2.4 (Assessment Methods); Section 2.5 (Outcomes); Section 2.4.4 (Outcome Assessment and Blinding)
**11**	Methods — Analysis	Qualitative and quantitative methods used to draw inferences from the data. Methods for understanding variation within the data, including the effects of time as a variable.	Section 2.6 (Statistical Analysis)
**12**	Methods — Ethical considerations	Ethical aspects of implementing and studying the intervention(s) and how they were addressed, including, but not limited to, formal ethics review and potential conflict(s) of interest.	Section 2.1 (Ethics Committee approval); Institutional Review Board Statement; Conflicts of Interest
**13**	Results — Results	Initial steps of the intervention(s) and their evolution over time (e.g., time-line diagram, flow chart, or table), including modifications made to the intervention during the project. Details of the process measures and outcomes. Contextual elements that interacted with the intervention(s). Observed associations between outcomes, interventions, and relevant contextual elements. Unintended consequences such as unexpected benefits, problems, failures, or costs associated with the intervention(s). Details about missing data.	Sections 3.1–3.7; Figures 1–3; Tables 1–3
**14**	Discussion — Summary	Key findings, including relevance to the rationale and specific aims. Particular strengths of the project.	Section 4.1 (Principal Findings); Section 5 (Conclusions)
**15**	Discussion — Interpretation	Nature of the association between the intervention(s) and the outcomes. Comparison of results with findings from other publications. Impact of the project on people and systems. Reasons for any differences between observed and anticipated outcomes, including the influence of context. Costs and strategic trade-offs, including opportunity costs.	Sections 4.2–4.5 (Discussion)
**16**	Discussion — Limitations	Limits to the generalizability of the work. Factors that might have limited internal validity such as confounding, bias, or imprecision in the design, methods, measurement, or analysis. Efforts made to minimize and adjust for limitations.	Section 4.6 (Limitations)
**17**	Discussion — Conclusions	Usefulness of the work. Sustainability. Potential for spread to other contexts. Implications for practice and for further study in the field. Suggested next steps.	Section 5 (Conclusions)
**18**	Other Information — Funding	Sources of funding that supported this work. Role, if any, of the funding organization in the design, implementation, interpretation, and reporting.	Funding statement (no external funding; APC by University of Medicine and Pharmacy of Craiova)

Supplementary Table S1. Department-level distribution of healthcare workers and study samples.

Department-level breakdown of the 71 healthcare workers (HCWs) enrolled across the three participating clinical departments, the distribution of professional roles, retention through the three study phases, and the totals of paired ATP–CFU samples and Semmelweis evaluations contributed by each phase. This descriptive breakdown complements Table 1 of the main manuscript and provides additional transparency regarding staffing composition and data contribution per department.

Characteristic	ICU	Hematology	Gynecology	Overall
**Total HCWs enrolled (n)**	**24**	**22**	**25**	**71**
**Professional role distribution**				
Attending physicians	4	4	4	12
Medical residents	1	1	1	3
Nurses	14	13	15	42
Nursing assistants	5	4	5	14
Withdrawals during study (n)	1	1	1	3
HCWs completing all three phases (n)	23	21	24	68
**Paired ATP–CFU samples (3 phases)**				**781**
Phase 1 (Baseline)	—	—	—	284
Phase 2 (Active intervention)	—	—	—	284
Phase 3 (Sustainability)	—	—	—	213
**Semmelweis evaluations**				
Protocol-based (Phases 2 + 3)	—	—	—	497
Targeted zone-level (Phase 3; 40 HCWs × 5 zones)	—	—	—	200

Abbreviations: *ICU, Intensive Care Unit; HCW, Healthcare Worker; ATP, Adenosine Triphosphate; CFU, Colony-Forming Units.*

Note. *As this is a single-arm before-after study, no between-group comparison is required. Phase-level sample counts are aggregated across the three departments and not stratified per department; per-department phase-level totals were not retained as separate variables in the analytic dataset.*
